# Validation of Reference Genes for Accurate Normalization of Gene Expression in *Lilium davidii* var. *unicolor* for Real Time Quantitative PCR

**DOI:** 10.1371/journal.pone.0141323

**Published:** 2015-10-28

**Authors:** XueYan Li, JinYun Cheng, Jing Zhang, Jaime A. Teixeira da Silva, ChunXia Wang, HongMei Sun

**Affiliations:** 1 Key Laboratory of Protected Horticulture of Education Ministry and Liaoning Province, College of Horticulture, Shenyang Agricultural University, Shenyang, P R China; 2 P. O. Box 7, Miki-cho post office, Ikenobe 3011–2, Kagawa-ken, 761–0799, Japan; Huazhong Agricultural University, CHINA

## Abstract

*Lilium* is an important commercial market flower bulb. qRT-PCR is an extremely important technique to track gene expression levels. The requirement of suitable reference genes for normalization has become increasingly significant and exigent. The expression of internal control genes in living organisms varies considerably under different experimental conditions. For economically important *Lilium*, only a limited number of reference genes applied in qRT-PCR have been reported to date. In this study, the expression stability of 12 candidate genes including *α-TUB*, *β-TUB*, *ACT*, *eIF*, *GAPDH*, *UBQ*, *UBC*, *18S*, *60S*, *AP4*, *FP*, and *RH2*, in a diverse set of 29 samples representing different developmental processes, three stress treatments (cold, heat, and salt) and different organs, has been evaluated. For different organs, the combination of *ACT*, *GAPDH*, and *UBQ* is appropriate whereas *ACT* together with *AP4*, or *ACT* along with *GAPDH* is suitable for normalization of leaves and scales at different developmental stages, respectively. In leaves, scales and roots under stress treatments, *FP*, *ACT* and *AP4*, respectively showed the most stable expression. This study provides a guide for the selection of a reference gene under different experimental conditions, and will benefit future research on more accurate gene expression studies in a wide variety of *Lilium* genotypes.

## Introduction

The genus *Lilium* is one of the most valuable commercial market flower bulbs in the world, mainly owing to its ornamental function as a cut flower or as a potted plant. Many *Lilium* species and cultivars are valued for their magnificent and showy flowers, more or less recurved tepals, distinctive fragrance, wide adaptability to soils and climates, and resistance to biotic stresses [1–4]. These characteristics have encouraged widespread biochemical, physiological and molecular biological studies of *Lilium* [5–8]. *Lilium davidii* var. *unicolor*, which originates from China, is famous for its economic and ornamental value resulting from its deep red and reflexed petals. It has long been thought of as the best edible lily in China since the scales are jade white and thick, glutinous and sweet, delicate, and without residue. Moreover, this species has also found uses in traditional medicine for purging lungs and dissolving phlegm, relieving stress and tranquilizing the body [9]. Consequently, molecular biological studies on this species have been increasingly performed in recent years [10,11]. However, since genomic resources for *Lilium* are still scarce, the analysis of *Lilium* genes, gene transcription and expression has been slow since most of the genes remain unknown.

Gene expression profiling is increasingly important to examine plant biological systems, especially to elucidate complex signaling as well as metabolic pathways that underlie developmental, biological and cellular processes [12–14]. Among the widely used methods to measure the levels of gene expression, it is undeniable that quantitative real time PCR (qRT-PCR) is a robust method for either identifying or monitoring gene expression profiles, and for assessing mRNA levels across different sample populations, with the following advantages: accuracy, sensitivity, specificity, ability to quantify, and reproducibility [15]. qRT-PCR can be used to directly compare mRNAs whose abundance differs widely and its accuracy is strongly affected by many variables, including the quality and quantity of mRNA templates, reverse transcription of mRNA, amplification efficiency, selection of reference genes and differences between cells or tissues [16,17]. Currently, these variations are minimized by normalizing gene expression to the expression of one or several reference genes [18,19]. However, the use of inadequate reference genes may result in interpretation errors; consequently, expression data may be misinterpreted [20]. Thus, appropriate reference genes are a prerequisite for qRT-PCR.

The reference gene, often termed the control gene, is assumed to be stably expressed, i.e., it should be constitutively expressed among different tissues and under different experimental parameters or treatments [13,21–23]. Some genes involved in basic cellular processes, primary metabolism and cell structure maintenance, are often used as normalizers [24]. Thus, the most traditional reference genes currently used in plant-related qRT-PCR studies include, among others, actin (*ACT*), eukaryotic initiation factor 1α (*eIF*), glyceraldehyde 3-phosphate dehydrogenase (*GAPDH*), tubulin (*TUB*), ubiquitin (*UBQ*), and 18S ribosomal RNA (*18S*). Nevertheless, the transcript levels of these most well-known and frequently used reference genes have been found to vary considerably depending on the developmental stage and experimental parameter [25–27]; and recently, an increasing number of reports have illustrated some novel reference genes are superior compared to those traditional reference genes in *Brassica juncea* [25], *Arabidopsis thaliana* [27], and other species. Indeed, the systemic use of putative reference genes without previous validation may lead to the misinterpretation of results. In recent years, the importance of validating reliable reference genes in each experimental condition prior to their use for normalization has been emphasized in many plant species [28–30].

qRT-PCR is commonly used to analyze gene expression in *Lilium*. To date, the traditional housekeeping genes *UBQ* [31], *GAPDH* [32,33], *18S* [21], and *ACT* [2,34,35] have been used as internal reference genes to standardize the expression profile of some genes. However, the relative stability of these and other potential reference genes in *Lilium* has not been validated in a range of experimental contexts, and this has constrained the wider use of qRT-PCR in *Lilium*.

In order to select the most appropriate reference genes for gene expression quantification by qRT-PCR, we examined different stress factors and developmental processes, including 29 diverse samples broadly categorized into seven distinct experimental sets. A total of 12 traditional and novel reference genes involved in different biological roles, *α-TUB* (alpha-tubulin), *β-TUB* (beta-tubulin), *ACT*, *AP4* (AP-4 complex subunit), *eIF*, *FP* (F-box family protein), *GAPDH*, *RH2* (DEAD box RNA helicase), *UBQ*, *UBC* (ubiquitin-conjugating enzyme), *18S*, and *60S* (60S ribosomal RNA), were evaluated using several statistical algorithms for the normalization of data. Some of the selected reference genes, which are commonly used as normalization factors in qRT-PCR analysis, such as *α-TUB*, *β-TUB*, *ACT*, *eIF*, *GAPDH*, *UBQ*, *UBC*, *18S*, and *60S*, and others (*AP4*, *FP* and *RH2*), showed high expression stability in other plant species and experimental conditions. In addition, the expression patterns of *DREB* (dehydration responsive element binding proteins) in *Lilium*, which regulates the plant’s response to different stresses, were investigated to illustrate the usefulness of the selected reference genes.

## Results

### Verification of primer specificity and PCR efficiency analysis

**Fig 1:**
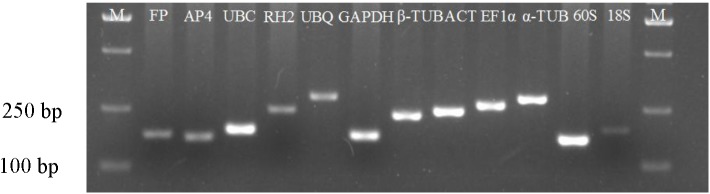
Amplification of the candidate reference genes from cDNA templates. Agarose gel electrophoresis showing amplification of a specific PCR product of the expect size for each gene.

In order to determine the specificity and efficiency of primers, 2% agarose gel electrophoresis analyses were performed to check the amplicons of the candidate reference genes derived from all templates. All the primers pairs amplified single fragments of the expected size (Fig 1). Sequencing analyses showed that all genes were 100% identical to their original genes deposited in the GenBank database (unpublished data); the sequences data of these genes are shown in S1 File. In addition, the specificity of the amplicons was confirmed by the presence of a single peak in melting curve analyses following qRT-PCR (S1 Fig), and no products were detected in negative controls. A standard curve was generated using 10-fold serial dilutions of pooled cDNA and the slopes of standard curves were used to check R^2^ values and PCR efficiency. The PCR efficiencies ranged from 95% to 105%, which are well within the acceptable range of 90–105% of qRT-PCR and suitable for further gene expression analysis by qRT-PCR (Table 1). In addition, the standard curves showed good linear relationships (R^2^ values ranged from 0.9910 to 0.9998) between Ct and the log-transformed copy numbers.

**Table 1:** Description of candidate reference genes from *Lilium davidii* var. *unicolor* for qRT-PCR analysis.

Internal gene	Gene name	Accession number	Primer sequence (5’-3’) forward/reverse	Amplicon length (bp)	Amplification efficincy (%)	Regression coefficient (R^2^)
*α-TUB*	Alpha-tubulin	KP861877	TGGCTTCACAGTCTATCCCTC/ GGGACAAGATTGGTCTGGAAC	282	98.96	0.9937
*β-TUB*	Beta-tubulin	KP861875	CTATGACATCTGTTTCCGCACTC/ AGCGATACTGTTGGGAGCCT	227	96.30	0.9990
*ACT*	Actin	KP861871	ATCTATGAGGGTTATGCTCTTCC/ CATCAGGCAGCTCGTAACTTC	241	100.91	0.9948
*AP4*	AP-4 complex subunit	KP861878	GATGGGGCTTCTTTATACGGT/ TCATTACAGCAAACTCTCCCTCT	163	104.31	0.9946
*eIF*	Eukaryotic initiation factor 1α	KP861874	TATGGTGAGCTTCCTGACAACGT/ TCACAAAGACAGTAACAACAGCGAT	265	97.09	0.9998
*FP*	F-box family protein	KP861876	TCGCCTACATCGCTAACC/ TTCCCAATAATCGCAAGACC	169	99.23	0.9961
*GAPDH*	Glyceraldehyde-3- phosphate dehydrogenase	gb|KP179417.1|	GCTGCAAGTTTCAACATTATTCC/ ATCCTCATCAGTATAACCAAGA	240	100.50	0.9910
*RH2*	DEAD box RNA helicase	KP861880	CCGAGACCAGTTCGTTCA/ ACAATAGGACCATCCCCAT	242	99.82	0.9994
*UBQ*	Ubiquitin	KP861873	TATGGTGGATTATCGGTTTCTACTG/ ACCACAGACTTTTTCAGTATCGCA	293	99.61	0.9959
*UBC*	Ubiquitin-conjugating enzyme	KP861872	GAGTGGAGCGTGACCATAAT/ CTGGTGGATGCAGAATTGAT	184	98.99	0.9985
*18S*	18S ribosomal RNA	gb|AY684927.1|	CGTTTCGGGCATGATTTGTGG/ TCGCATTTCGCTACGTTCTTC	183	96.82	0.9963
*60S*	60S ribosomal RNA	KP861879	GCAAAGGCTGTCAAAAATCAGGTAG/ ATAACCCACAAACTAATAGCCCTGC	156	98.39	0.9916

### Expression levels of candidate reference genes

**Fig 2:**
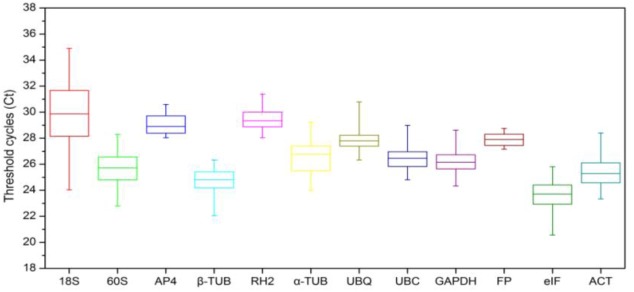
Expression levels of 12 candidate reference genes in all samples. Expression data displayed as Ct values for each reference gene in all *Lilium* samples. The line across each box depicts the median. The box indicates the 25/75 percentiles while whisker caps represent 1/99 percentiles.

An overview of the expression stability of the 12 candidate reference genes from different treatments across all samples is displayed in Fig 2. The Ct values of reference genes showed a range of variation from 20 to 35 cycles, and most Ct values were between 22 and 29 cycles. The *18S* gene was the least abundant with the Ct in the range of 26–32, while *eIF* displayed the highest expression level with Ct values of less than 25 cycles. The calculated coefficient of variance (CV) of the Ct values provides an indication of the expression stability of a particular gene. The narrower the range of the Ct values, the more stably the given gene was expressed in different tissue samples. Among the 12 candidate reference genes examined in this study, *18S* showed much greater variation in expression levels than other genes with a CV value greater than 6 cycles, whereas *FP*, with a minimal CV of 0.42, remained relatively constant in all samples.

### GeNorm analysis

**Fig 3:**
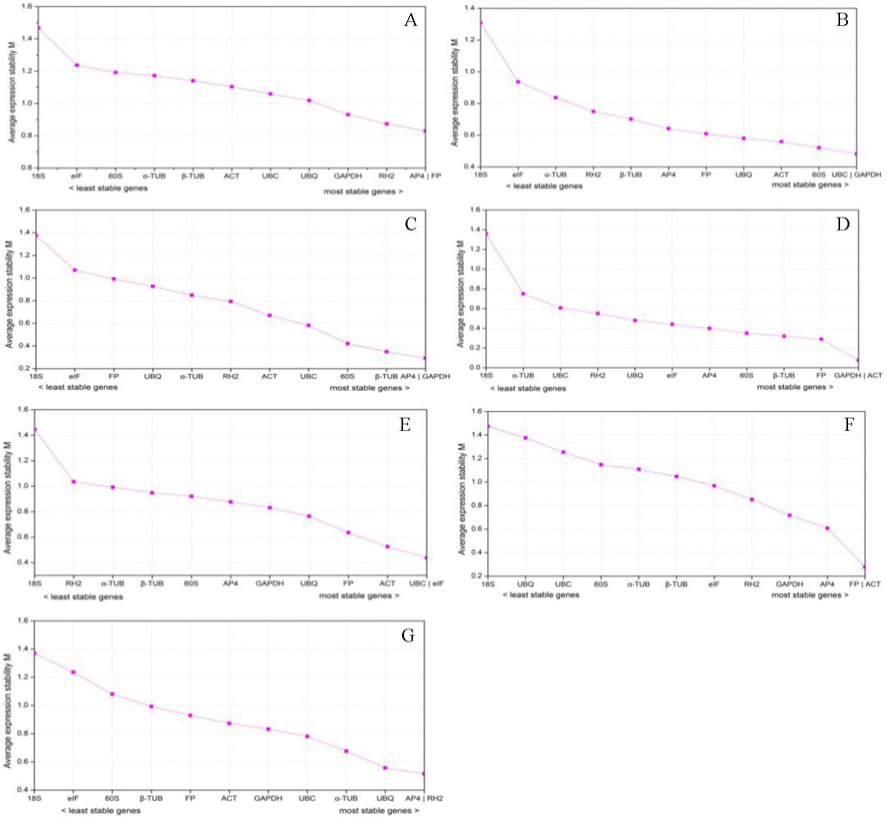
Average expression stability values (M) of the 12 candidate reference genes as calculated by geNorm. (A) All 29 samples, (B) different organs, (C) leaves in different developmental processes, (D) scales in different developmental processes, (E) leaves under stress treatments, (F) scales under stress treatments, (G) roots under stress treatments.

GeNorm software was employed to determine the expression stability of the selected genes as described by [36]. The raw Ct values were transformed into relative expression levels, and the average expression stability value (M) was ranked. According to the geNorm applet, the least stable reference gene shows the highest M value while the most stable gene presents the lowest M. Besides, Hellemans [36] recommended a stability measure threshold lower than 1.5 to ensure that only the most stable genes are selected. The results obtained are shown in Fig 3, in which we analyzed data from 7 sets of treatments. The M value of all tested genes was less than 1.5. Considering all 29 tissues (set A), the *AP4* and *FP* genes were ranked as most stable, both with an M value of 0.830. Among the different organs (set B), *UBC* and *GAPDH* performed well, displaying the lowest M value (0.482) while *18S* presented the highest M value (1.309). For different developmental processes, the most stable genes were *AP4* as well as *GAPDH* with the lowest M value in leaves (set C), and *ACT* as well as *GAPDH* in scales (set D). Under stress treatments, *UBC* and *eIF* were the most highly ranked with an M value of 0.438 in leaves (set E), *FP* and *ACT* were the most stably expressed genes in scales (set F), and *AP4* and *RH2* were expressed more stably than other genes in roots (set F). In all seven experimental sets, *18S* was the least stable gene.

**Fig 4:**
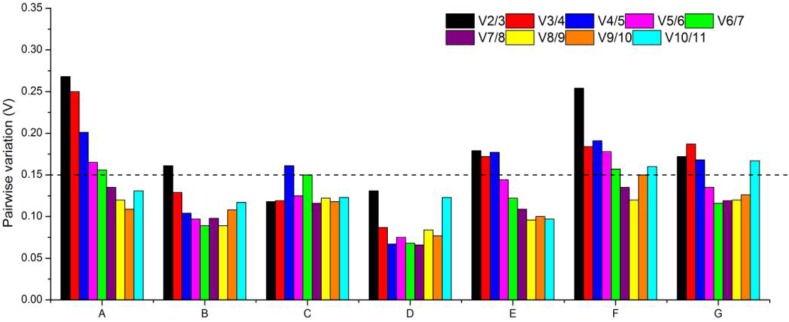
Pairwise variation (V) calculated by geNorm to determine the optimal number of reference genes. (A) all 29 samples, (B) different organs, (C) leaves in different developmental processes, (D) scales in different developmental processes, (E) leaves under stress treatments, (F) scales under stress treatments, (G) roots under stress treatments.

The optimum number of reference genes required for accurate normalization needs to be ascertained according to certain experimental conditions because normalization with a single reference gene can sometimes produce significant errors (37). Thus, pairwise variation (V) was also applied to assess the optimal number of reference genes required for reliable normalization. A threshold V_n_/V_n+1_ value of 0.15 was adopted to determine whether the inclusion of an additional reference gene was necessary [37]. As shown in Fig 4, the ideal number of reference genes may be different for a distinct set of samples. For instance, a V2/V3 score lower than 0.15 was achieved both in leaves and scales under different developmental processes (sets C and D), indicating that the combination of two stable reference genes would be sufficient for the normalization of gene expression. In different organs (set B), the addition of a third gene was necessary to normalize gene expression (V3/V4 value was 0.129). When all samples were pooled for analysis (set A), and leaves, scales as well as roots under stress treatments (sets E, F and G), more than five genes were sufficient for normalization. However, adding too many reference genes will increase the instability, and also the complexity of the experimental work [38]. Consequently, only one reference gene can be applied, resulting in accurate normalization.

### NormFinder analysis

The stability of the reference gene was further analyzed by NormFinder, which takes inter- and intra-group variations into account and combines both results into a stability value for each candidate reference gene. Candidate reference genes with a lower average expression stability value are more stably expressed. The NormFinder outputs are shown in Table 2. The top-ranked candidates also differed in different data sets using this method of analysis. In all samples (set A), the two most stable genes calculated by NormFinder were the same as those determined by geNorm. In different organs (set B), *UBQ* performed better than other genes, and *ACT*, *FP* and *60S* ranked among the top positions. In different developmental processes, *ACT* was the top rank for leaves (set C), while *GAPDH* showed the most stable transcriptional expression in scales (set D). Taking into account the stress treatments, *FP*, *ACT* or *AP4* were ranked as first in leaves (set E), second in scales (set F) and third in roots (set G), respectively. Additionally, *18S* was the least stable gene in all seven data sets, corresponding with the result analyzed by the distinct statistical algorithm geNorm analysis.

**Table 2:** Ranking of reference genes and their expression stability values calculated using NormFinder.

Rank	Total (A)		Organs (B)		Leaves in developmental process (C)		Scales in developmental process (D)		Leaves under stress (E)		Scales under stress (F)		Roots under stress (G)	
	Gene name	Stability value	Gene name	Stability value	Gene name	Stability value	Gene name	Stability value	Gene name	Stability value	Gene name	Stability value	Gene name	Stability value
1	*FP*	0.496	*UBQ*	0.276	*ACT*	0.221	*GAPDH*	0.038	*FP*	0.230	*ACT*	0.296	*AP4*	0.378
2	*AP4*	0.547	*ACT*	0.286	*AP4*	0.435	*ACT*	0.038	*AP4*	0.405	*FP*	0.536	*UBC*	0.540
3	*GAPDH*	0.724	*FP*	0.293	*RH2*	0.443	*FP*	0.046	*60S*	0.575	*AP4*	0.699	*RH2*	0.541
4	*β-TUB*	0.839	*60S*	0.304	*β-TUB*	0.589	*β-TUB*	0.046	*UBQ*	0.736	*GAPDH*	0.758	*α-TUB*	0.667
5	*UBC*	0.897	*GAPDH*	0.516	*UBQ*	0.689	*60S*	0.140	*UBC*	0.739	*eIF*	0.780	*FP*	0.678
6	*RH2*	0.908	*UBC*	0.631	*α-TUB*	0.699	*UBQ*	0.485	*GAPDH*	0.743	*β-TUB*	0.952	*GAPDH*	0.812
7	*60S*	0.929	*β-TUB*	0.650	*GAPDH*	0.700	*AP4*	0.526	*ACT*	0.782	*RH2*	1.071	*UBQ*	0.863
8	*UBQ*	0.962	*AP4*	0.672	*60S*	0.981	*eIF*	0.564	*β-TUB*	0.848	*α-TUB*	1.138	*β-TUB*	0.902
9	*ACT*	0.968	*RH2*	0.736	*FP*	1.061	*RH2*	0.681	*RH2*	0.907	*60S*	1.160	*ACT*	0.905
10	*α-TUB*	0.981	*α-TUB*	1.113	*UBC*	1.108	*UBC*	1.003	*α-TUB*	0.983	*UBC*	1.408	*60S*	1.231
11	*eIF*	1.251	*eIF*	1.348	*eIF*	1.444	*α-TUB*	1.435	*eIF*	1.022	*UBQ*	1.664	*18S*	1.838
12	*18S*	2.474	*18S*	3.102	*18S*	2.830	*18S*	4.383	*18S*	3.435	*18S*	1.704	*eIF*	1.857

### BestKeeper analysis

The BestKeeper program is another software tool to analyze the stability of a candidate reference gene based on the standard deviation (SD) of the Ct values. Reference genes are identified as the most stable genes when they exhibit the lowest SD. In this study (Table 3), BestKeeper analysis revealed that *FP* had the lowest SD values in all samples (set A) and leaves under three stresses (set E). However, *GAPDH* showed stable expression in different organs (set B). For different developmental processes, *RH2* or *eIF* were expressed more stably than the other reference genes in leaves (set C) and scales (set D), respectively. As for scales (set F) and roots (set G) under three stress treatments, the top two stable expressed genes were the same as NormFinder, but were ranked in a different order.

**Table 3:** Ranking of reference genes and their expression stability values calculated using BestKeeper.

Rank	Total (A)		Organs (B)		Leaves in developmental process (C)		Scales in developmental process (D)		Leaves under stress (E)		Scales under stress (F)		Roots under stress (G)	
	Gene name	Stability value	Gene name	Stability value	Gene name	Stability value	Gene name	Stability value	Gene name	Stability value	Gene name	Stability value	Gene name	Stability value
1	*FP*	0.424	*GAPDH*	0.298	*RH2*	0.173	*eIF*	0.218	*FP*	0.212	*FP*	0.169	*UBC*	0.368
2	*AP4*	0.634	*ACT*	0.325	*ACT*	0.208	*β-TUB*	0.272	*UBC*	0.428	*ACT*	0.298	*AP4*	0.556
3	*RH2*	0.682	*60S*	0.334	*α-TUB*	0.291	*FP*	0.300	*UBQ*	0.489	*GAPDH*	0.555	*FP*	0.626
4	*UBC*	0.696	*UBC*	0.338	*UBQ*	0.447	*AP4*	0.318	*AP4*	0.580	*RH2*	0.734	*GAPDH*	0.632
5	*UBQ*	0.703	*UBQ*	0.382	*FP*	0.528	*60S*	0.319	*ACT*	0.618	*AP4*	0.781	*ACT*	0.696
6	*GAPDH*	0.708	*FP*	0.430	*β-TUB*	0.576	*GAPDH*	0.347	*60S*	0.629	*β-TUB*	0.943	*RH2*	0.720
7	*β-TUB*	0.842	*AP4*	0.461	*AP4*	0.630	*ACT*	0.399	*eIF*	0.702	*eIF*	1.002	*α-TUB*	0.822
8	*ACT*	0.874	*RH2*	0.551	*GAPDH*	0.745	*UBC*	0.484	*GAPDH*	0.824	*UBC*	1.029	*UBQ*	0.831
9	*eIF*	0.972	*β-TUB*	0.578	*60S*	0.823	*UBQ*	0.516	*RH2*	0.844	*α-TUB*	1.122	*β-TUB*	0.906
10	*α-TUB*	1.074	*eIF*	0.855	*UBC*	0.859	*RH2*	0.668	*α-TUB*	0.862	*UBQ*	1.210	*eIF*	1.039
11	*60S*	1.100	*α-TUB*	0.964	*eIF*	1.235	*α-TUB*	1.258	*β-TUB*	0.923	*60S*	1.289	*60S*	1.126
12	*18S*	2.024	*18S*	2.019	*18S*	1.881	*18S*	3.52	*18S*	2.738	*18S*	1.340	*18S*	1.604

### Comparative ΔCt

The ΔCt method assesses gene expression stability by calculating pair-wide differences of Ct (ΔCt). According to the ΔCt method (Table 4), *FP* was the most highly ranked gene in all samples (set A), indicating that it is the most stable gene. For organs (set B) and leaves in different developmental processes (set C), *ACT* was the most stable gene while *60S* performed better than other candidate genes in scales in different developmental processes (set D). Similar to BestKeeper, the top two stable genes in leaves under stress (set E) were *FP* and *UBC*. In accordance with NormFinder, *ACT* and *FP* were the top ranked genes in scales (set F). In addition, *AP4* was most stably expressed in roots under three stress conditions (set G).

**Table 4:** Ranking of reference genes and their expression stability values calculated using ΔCt.

Rank	Total (A)		Organs (B)		Leaves in developmental process (C)		Scales in developmental process (D)		Leaves under stress (E)		Scales under stress (F)		Roots under stress (G)	
	Gene name	Stability value	Gene name	Stability value	Gene name	Stability value	Gene name	Stability value	Gene name	Stability value	Gene name	Stability value	Gene name	Stability value
1	*FP*	1.18	*ACT*	0.94	*ACT*	1.04	*60S*	0.90	*FP*	1.04	*ACT*	1.12	*AP4*	1.06
2	*AP4*	1.21	*UBQ*	0.96	*β-TUB*	1.06	*GAPDH*	0.91	*UBC*	1.16	*FP*	1.18	*RH2*	1.10
3	*GAPDH*	1.28	*60S*	0.99	*AP4*	1.08	*FP*	0.93	*AP4*	1.19	*AP4*	1.27	*UBC*	1.13
4	*β-TUB*	1.34	*FP*	1.04	*RH2*	1.12	*ACT*	0.94	*60S*	1.23	*GAPDH*	1.30	*α-TUB*	1.16
5	*UBC*	1.35	*GAPDH*	1.04	*GAPDH*	1.12	*β-TUB*	0.96	*ACT*	1.23	*eIF*	1.30	*FP*	1.20
6	*RH2*	1.39	*UBC*	1.07	*α-TUB*	1.21	*AP4*	0.98	*UBQ*	1.23	*β-TUB*	1.42	*GAPDH*	1.24
7	*60S*	1.41	*AP4*	1.11	*60S*	1.24	*eIF*	1.07	*GAPDH*	1.29	*RH2*	1.46	*UBQ*	1.28
8	*UBQ*	1.42	*β-TUB*	1.16	*UBQ*	1.29	*RH2*	1.13	*β-TUB*	1.32	*α-TUB*	1.50	*ACT*	1.31
9	*ACT*	1.42	*RH2*	1.20	*UBC*	1.37	*UBQ*	1.13	*eIF*	1.33	*60S*	1.52	*β-TUB*	1.33
10	*α-TUB*	1.43	*α-TUB*	1.45	*FP*	1.44	*UBC*	1.25	*RH2*	1.41	*UBC*	1.72	*60S*	1.56
11	*eIF*	1.59	*eIF*	1.59	*eIF*	1.62	*α-TUB*	1.71	*α-TUB*	1.42	*UBQ*	1.92	*eIF*	2.02
12	*18S*	2.62	*18S*	3.17	*18S*	2.90	*18S*	4.41	*18S*	3.51	*18S*	1.96	*18S*	2.04

### Overall ranking order and selection of candidate genes by RefFinder

The four software tools which were employed to analyze the data gave different results and different statistical stability values for each gene. RefFinder applet was used to arrange the comprehensive results which integrated the data of the four statistical approaches to compare and rank the potential reference genes. The results of the aggregate order showed that *FP* was optimal for transcriptome analysis in all samples (set A) and in leaves under three stress treatments (set E). For organs, leaves in different developmental processes (set C) and scales under three stress treatments (set F), *ACT* presented the most stable expression. *GAPDH* was the best candidate gene in scales in different developmental processes (set F), while *AP4* was the best in roots under three stress conditions (set G).

### Reference gene validation

**Fig 5:**
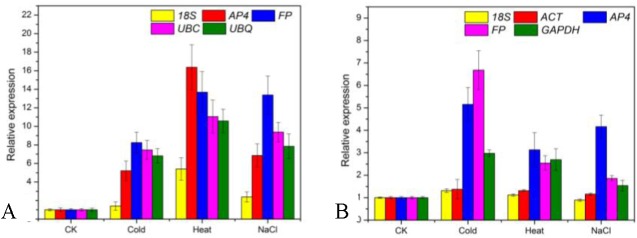
Relative quantification of *DREB* to validate candidate inference genes of *Lilium unicolor* var. *davidii* under abiotic stresses. (A) Expression levels of *DREB* in leaves using identified stable reference genes (*AP4*, *FP*, *UBC*, and *UBQ*) and least stable reference genes (*18S*). (B) Expression levels of *DREB* in scales using identified stable reference genes (*ACT*, *AP4*, *FP*, and *GAPDH*) and least stable reference genes (*18S*). Unstressed plants were used as the control (CK).

To detect the effect of the reference genes on the outcome of a practical experiment, the relative expression patterns for *DREB* were analyzed using different reference genes. *DREB* is an important transcription factor that imparts stress endurance to plants and plays key roles in providing tolerance to heat, dehydration, wounding and salt stress [39–41]. In *Lilium*, it has been reported that *DREB* can induced by dehydration, cold and salt stress [42], and the transformed *DREB* gene can enhance tolerance to high temperature [43]. As suggested by the geNorm approach, more than five genes were sufficient for normalization of leaves as well as scales under stress treatments (sets E and F). Consequently, only one reference gene was further used as an internal control. The most stable genes in leaves under stress were *FP*, *UBC*, *UBQ*, and *AP4*, the most stable reference genes in scales under stress were *ACT*, *AP4*, *FP*, and *GAPDH*, while the *18S* reference gene was identified as the least stable gene in both sets. The expression of *DREB* increased after cold, heat, and NaCl treatments, with a relative expression >1 (Fig 5). However, DREB was expressed at a lower level when using the least stable reference *18S* gene as the internal control, especially in scales treated with NaCl (less than 1 obtained in the control). Thus, the use of unsuitable reference genes may lead to an over- or underestimation of relative transcript abundance. These results reinforce the importance of validating reference genes prior to experimental applications.

## Discussion

qRT-PCR has become a powerful technique for detecting and quantifying the gene expression patterns of particular genes in distinct biological samples, because of its high throughput, efficiency, and reliability [15]. Until recently, it has often been assumed that the choice of stably expressed reference genes for normalization is paramount to accurate interpretation of the results [12,44]. As no single gene has stable expression under every experimental condition, it is advisable and necessary to validate the expression stability of candidate reference genes by taking into account variation in samples, developmental status, and experimental treatments [12,27,44]. The evaluation of expression stability of potential reference genes has been addressed under special conditions for species such as *Arabidopsis thaliana* [24], bamboo (*Phyllostachys edulis*) [28], *Brassica juncea* [25], cucumber (*Cucumis sativus* L.) [45], longan (*Dimocarpus longan* Lour.) [46], olive (*Olea europaea*) [47], peach (*Prunus persica* L. Batsch) [48], *Pyrus pyrifolia* [49, 50], *Populus* [51], rice (*Oryza sativa*) [52], ryegrass (*Lolium perenne* L.) [53], strawberry (*Fragaria*×*ananassa* Duch) [29], tomato (*Solanum lycopersicum*) [54], and tung (*Vernicia fordii* Hemsl.) [55]. However, only limited attempts at reference gene validation have been reported for ornamental plants, including *Chrysanthemum lavandulifolium* [56], *Chrysanthemum* [57], *Petunia* [58], and water lily (*Nymphaea* spp.) [59]. Transcriptional stability is dependent on the tested material and on the experimental treatments. To our knowledge, there is no information in the literature regarding the choice of reference genes for gene expression studies in *Lilium*. The detailed analyses in this study included a broad spectrum of samples: different sample tissues, different developmental processes, and abiotic stress treatments.

Several calculation algorithms are available to investigate the expression stability of proposed reference genes, and it is assumed that a comparison of different algorithms allows for better evaluation [48]. Therefore, the present work employed four software packages, geNorm, NormFinder, BestKeeper, and ΔCt, to comprehensively investigate the transcriptional stability of 12 candidate genes (including nine traditional housekeeping genes and three novel candidate reference genes) in 29 diverse samples of *Lilium*, divided into seven experimental sets. Some reports suggested that applying different analysis software would result in different validation results in the same tissue or treatment, due to their distinct statistical algorithms and analytical procedures [44,60,61]. Two of the reference genes *FP* and *AP4* were classified as the most stably transcribed among all samples (set A) when the four algorithms were employed (Fig 3, Tables 2–4). The top two positions (*GAPDH* and *ACT*) in scales in different developmental processes (set D) predicted by geNorm (Fig 3) were similar to those determined by NormFinder (Table 2). The top two positions (*FP* and *UBC*) in leaves under stress treatments (set E) generated by BestKeeper (Table 3) were similar to those determined by ΔCt (Table 4). Therefore, it is necessary to validate the expression stability of the reference gene under specific experimental conditions prior to its use for normalization.

Some of the novel candidate reference genes selected in the current study performed better than the traditional housekeeping genes under specified conditions. In rubber tree (*Hevea brasiliensis*), *RH2* was identified as the most stable reference gene across individual trees [62]. Previous studies on the selection of reference genes in cotton (*Gossypium hirsutum* L.) also identified *FP* as the most stable reference gene in floral verticils [44]. A similar result for was observed by [63], whereby the expression patterns of *FP* appeared to be the most stable in virus-infected *Nicotiana bethamiana*. In cucumber, *FP* also showed stable expression [45]. Similarly, we found that *FP* was the most stable reference gene in all samples and in leaves under three stress conditions.

The expression stability of traditional housekeeping genes like *α-TUB*, *β-TUB*, *ACT*, *eIF*, *GAPDH*, *UBQ*, *UBC*, *18S*, and *60S* was tested in this study. It was found that *ACT* and *GAPDH* showed more stable expression than other genes (Table 5), which was in accordance with observations made in *Populus* [50], *Pyropia yezoensis* [63], and eggplant (*Solanum melongena* L.) [35]. Our data demonstrates that *α-TUB*, *β-TUB*, *eIF*, and *60S* ranked in a middle position among all seven sets of experiments. In a previous study, *SiTUB* was shown to be suitable for sesame vegetative tissue development [30], *eIF* emerged as the most appropriate reference gene in logan (*Dimocarpus longan* Lour.) somatic embryogenesis [46]. Previously, *18S* has been considered to be one of the worst reference genes for assessing gene expression in many plant tissues and under different conditions [30,46,62]. Coincidentally, *18S* performed poorly in the present study. In contrast, *18S* has proved to be the most stable reference gene in strawberry (*Fragaria*×*ananassa* Duch) [29] and rice (*Oryza sativa* L.) [52]. This result confirms the vital necessity to evaluate reference genes according to the studied experimental conditions.

**Table 5:** Ranking of candidate reference genes in decreasing order of expression stability calculated by RefFinder.

Rank	Total (A)		Organs (B)		Leaves in developmental process (C)		Scales in developmental process (D)		Leaves under stress (E)		Scales under stress (F)		Roots under stress (G)	
	Gene name	Stability value	Gene name	Stability value	Gene name	Stability value	Gene name	Stability value	Gene name	Stability value	Gene name	Stability value	Gene name	Stability value
1	*FP*	1.00	*ACT*	2.00	*ACT*	1.86	*GAPDH*	1.86	*FP*	1.41	*ACT*	1.19	*AP4*	1.19
2	*AP4*	1.68	*GAPDH*	2.24	*AP4*	2.55	*ACT*	2.74	*UBC*	2.51	*FP*	1.41	*UBC*	2.34
3	*GAPDH*	3.83	*UBQ*	2.66	*RH2*	3.03	*FP*	3.00	*UBQ*	3.94	*AP4*	3.41	*RH2*	2.45
4	*RH2*	4.24	*60S*	3.22	*β-TUB*	3.46	*60S*	3.34	*AP4*	3.98	*GAPDH*	3.72	*α-TUB*	4.60
5	*UBC*	4.95	*UBC*	3.46	*GAPDH*	4.09	*β-TUB*	3.56	*ACT*	4.79	*RH2*	5.60	*FP*	4.95
6	*β-TUB*	5.47	*FP*	4.56	*α-TUB*	5.42	*eIF*	4.45	*60S*	5.26	*eIF*	5.69	*GAPDH*	5.42
7	*UBQ*	6.32	*AP4*	7.24	*UBQ*	6.16	*AP4*	5.63	*eIF*	5.46	*β-TUB*	6.24	*UBQ*	5.86
8	*ACT*	8.21	*β-TUB*	7.97	*60S*	6.70	*UBQ*	7.90	*GAPDH*	6.48	*α-TUB*	8.24	*ACT*	7.09
9	*60S*	8.57	*RH2*	8.74	*FP*	8.19	*RH2*	8.97	*RH2*	7.38	*UBC*	9.46	*β-TUB*	8.74
10	*α-TUB*	9.74	*α-TUB*	10.24	*UBC*	8.19	*UBC*	9.46	*β-TUB*	8.92	*60S*	9.46	*60S*	10.24
11	*eIF*	10.46	*eIF*	10.74	*eIF*	11.00	*α-TUB*	11.00	*α-TUB*	10.24	*UBQ*	10.74	*EIF*	10.98
12	*18S*	12.00	*18S*	12.00	*18S*	12.00	*18S*	12.00	*18S*	12.00	*18S*	12.00	*18S*	11.74

To test the suitability of candidate reference genes, the *DREB* gene was used in this study. *DREB* plays a crucial role in providing tolerance to abiotic stresses. In *Brassica juncea* [25], peanut (*Arachis hypogaea* L.) [64] and pearl millet (*Pennisetum glaucum* (L.) R. Br.) [65], *DREB* increased in stress treatments when normalized using selected reference genes, although at different levels. In pearl millet, a strong bias in the relative pattern of *DREB* was obtained when the least stable gene (*UBQ5*) was used for normalization. Similarly, an inaccurate transcriptional profile (the relative expression level of *DREB* was less than 1) was found in scales treated after NaCl using the least stable reference gene *18S* (Fig 5).

Taken together, the data obtained in previous studies and in the present research confirms the need to validate reference genes under different experimental conditions. The genes evaluated in this study will be very useful for further gene expression analysis to explore the molecular mechanisms related to plant development, quality formation (bulbs or cut flowers), environmental responses as well as the improvement of genetic traits in *Lilium*. To our knowledge, this is the first systematic study of the expression stability of reference genes across such a large number of samples under varied developmental processes and stress treatments in *Lilium*. Moreover, this study provides useful guidelines for the selection of reference genes in other Liliaceae species.

## Methods

### Plant materials

*Lilium davidii* var. *unicolor*, grown at the horticultural research base of Shenyang Agricultural University (N41°50ʹ, E123°34ʹ), was used for the experiments. No specific permits were required by the scientific research base to select samples. The research base is not privately-owned and the field studies did not involve any endangered or protected species.

For field development, lily bulbs (with a circumference of 14 cm) were planted on April 16 at a planting density of 15 cm inter-row spacing and 20 cm inter-line distance. Soil thickness above the bulbs was 10 cm. Lily plants received standard horticultural practices and disease as well as insect control. From the bud stage (May 30) to 20 days after flowering (July 17), the foliage was sprayed with 0.3% monopotassium phosphate and 1% urea every 3 days. About a month after the bud stage, the alabastrums were nearly 1.5 cm long and were about to bloom, and the circumference of bulblets formed on the stems in soil was almost 1 cm.

For scale cutting propagation, healthy external scales without any damage were carefully removed from the base of mother bulbs, washed in running water to remove dirt, surface sterilized by immersing in 0.01% potassium permanganate solution for 20 min, and then washed with distilled water three times using an in-house protocol. After surface sterilization, scales (three biological replicates, 150 scales in each) were embedded concave upward *ex vitro* into pre-sterilized (180°C for 5 h) wet peat substrate (XinYuan Gardening Resources Ltd., Liaoning, PR China) with 60% relative humidity, at 90 scales/300 cm^2^ (60 cm × 5 cm). Propagules were placed into perforated plastic bags (60 cm × 90 cm) and then incubated at 25°C under a photosynthetic photon flux (PPFD) of 50 μmol m^-2^ s^-1^. About 2 months after scale cuttings, the leaves that formed from the bulblets (with a circumference of 1 cm) were about 5 cm in length.

For tissue culture, aseptic seedlings were induced from scales according to Xu [66]. Rooted *Lilium* plantlets with bulblets having a 1 cm circumference were then cultured in Murashige and Skoog (MS) [67] medium supplemented with 60 g/L sucrose and 7 g/L agar. Embryogenic callus was induced from the scales of aseptic seedlings and sub-cultured every 30 days. For stress treatments, aseptic seedlings with bulblets having a 1 cm circumference were subjected to cold (4°C), heat (42°C), and salt (200 mM NaCl) treatments for 12 h and 36 h (short-term vs long-term stress). All cultures were placed under a photosynthetic photon flux (PPFD) of 50 μmol m^-2^ s^-1^ using fluorescent light with a 14-h photoperiod.

### Experimental design

A total of 29 samples were collected under different stresses and developmental stages. The expression stability of candidate reference genes was analyzed in the following seven experimental sets. All experimental sets were processed in sets of three replicates each. Sampled tissues were flash frozen in liquid nitrogen and stored at -80°C until further processing.

The first experimental set A (all) was composed of all samples.

In experimental set B (plant organs), leaves, mother bulbs, bulblets on stem (with 1 cm circumference), basal roots, and petals sampled from three different plants with a 1.5 cm alabastrum in the field, as well as embryogenic callus *in vitro*, were sampled.

Samples from the third and fourth experimental sets C and D represent leaves and scales in different developmental processes, respectively. There were three main developmental processes: field development, scale cutting propagation, and tissue culture. The leaves from set C and the scales from set D were collected from leaves and bulblets on the stem from plants with a 1.5 cm alabastrum in the field, 60 d after embedding scale cuttings, and aseptic seedlings with bulblets having a 1 cm circumference *in vitro*.

The fifth to seventh experimental sets E, F, and G represent leaves, scales and roots from three different aseptic seedlings in stress treatments, respectively.

### Total RNA extraction first-strand cDNA synthesis

Frozen samples were ground to a fine power in liquid nitrogen using a mortar and pestle sterilized at 180°C for 8 h. Total RNA was extracted from the collected tissues following Li [68]. To eliminate any traces of genomic DNA contamination after RNA extraction, DNase I (Tiangen, Beijing, China) was used as recommended by the manufacturer. The integrity of the RNA was assessed on a 1% (w/v) agarose (Invitrogen, CA, USA) gel. RNA concentration and the 260/280 as well as 260/230 absorbance ratios were determined using an Infinite^®^ 200 PRO (Tecan, Männedorf, Switzerland). First-strand cDNA was synthesized from 1 μg of DNase I-treated RNA using anchored-oligo (dT)s primers according to the manufacturer’s instructions (Promega, Madison, USA). Before each qRT-PCR stage, cDNA products were diluted five-fold prior to use.

### Selection of reference genes and primer design

The 12 candidate genes including nine traditional housekeeping genes (*α-TUB*, *β-TUB*, *ACT*, *eIF*, *GAPDH*, *UBC*, *UBQ*, *18S*, and *60S*) and three novel reference genes (*AP4*, *FP*, and *RH2*) were selected from the transcriptome of *Lilium davidii* var. *unicolor* bulblet development [10]. The gene sequences (except *18S*) were obtained and deposited in the GenBank database (accession numbers are listed in Table 1). The novel reference genes were homologous with newly identified stable reference genes in previous studies (44,61,62). Primer pairs were designed using Primer Premier 5.0 software (http://www.premierbiosoft.com/) with melting temperatures (*Tm*) of 55–65°C, a primer length of 17–25 bp, and an amplicon length ranging from 100 to 300 bp (Table 1). To ensure target specificity, gene sequences were blasted against the NCBI database to determine cross homology with other sequences. Primer specificities were confirmed by 2% agarose gel electrophoresis for a single product giving the expected size as described in Table 1.

### qRT-PCR conditions and PCR efficiency

Experiments were performed in 96-well PCR plates (Corning, NY, USA) with an ABI 7500 Real-Time PCR System (Life Technologies, CA, USA) using SYBR^®^ green (CWBIO, Beijing, China). Quantitative real-time PCR was carried out in a total volume of 20 μl containing 0.8 μl of template, 0.2 μM of each primer combination, and 1× UltraSYBR Mixture (with ROX). The following amplification program was used: denaturation at 95°C for 10 min, 44 cycles of amplification (95°C for 30 s, 60°C for 30 s, 68°C for 1 min) and a melting curve program (95°C for 15 s, 60°C for 1 min, 95°C for 30 s, 60°C for 15 s). For the negative control for each primer pair, no template was added to the reaction mixture, which resulted in no detectable fluorescence signal from the reaction. All reactions were performed in three biological and technical replicates. The standard curve of a 10-fold dilution series from a pool of cDNAs was made in triplicate to calculate the gene-specific PCR efficiency (E = 10^(-1/slope)^-1) and regression coefficient (R^2^).

### Determination of reference gene expression stability using geNorm, NormFinder, BestKeeper, and ΔCt

Reference gene transcript abundance in all samples was determined by the Ct value. Four statistical approaches were applied to assess the stability of the candidate reference genes: geNorm v3.5 (http://medgen.ugent.be/jvdesomp/genorm/) [36], NormFinder (http://www.mdl.dk/publications normfinder.htm) [69], BestKeeper (http://bioinformatics.gene-quantification.info/bestkeeper.html) [70], and the ΔCt method [71]. For geNorm and NormFinder algorithms, the raw Ct values must be transformed into relative quantities (only Ct<40 were used for analysis). The maximum expression level (i.e. the lowest Ct value) of each gene was used as a control and was set at 1. Relative expression levels were then calculated from Ct values using the formula 2^-ΔCt^, in which ΔCt = each corresponding Ct value minus the minimum Ct value. The resulting data were further analyzed using the geNorm and NormFinder algorithm. geNorm software also calculates the optimal number of reference genes needed for normalization. The data obtained from each biological replicate were analyzed separately. An additional tool, RefFinder (http://www.leoxie.com/referencegene.php) was used to compare and rank the stability of candidate genes integrating the outcomes of the above four statistical algorithms.

### Validation of reference gene analysis

One gene coding for the DREB2 family was used to validate the selected reference gene (GeneBank No. KP866251), whose main response is to salinity, heat and dehydration stress; however, in some monocotyledonous plants, DREB2 also responds to cold stress [72,73]. The forward primer is 5ʹ-TCCACCCGTCAACAACA-3ʹ and the reverse primer is 5ʹ -GTTGAGCCGAGCGAAGT-3ʹ. Primer design and qRT-PCR reactions were followed as mentioned before. As determined by the RefFinder algorithm (Table 5), the most stable genes selected in leaves (*FP*, *UBC*, *UBQ*, and *AP4*) as well as in scales (*ACT*, *FP*, *AP4*, and *GAPDH*), and the least stable gene both in leaves and scales (*18S*) under three stress treatments were used as internal reference genes. Aseptic seedlings not exposed to any abiotic stress were used as the control. The relative expression levels of the target gene in leaves and scales under three stress treatments were represented as relative expression (2^-ΔΔCt^).

## Supporting Information

S1 Fig Specificity of qRT-PCR amplification. Dissociation curves of the 12 candidate reference genes after the qRT-PCR reactions, all showing a single peak.
(TIF)

S1 File Sequencing data of 12 candidate reference genes. (DOC)
