# Supplementary figures and images for: Validation of Reference Genes for Accurate Normalization of Gene Expression in Lilium davidii var. unicolor for Real Time Quantitative PCR
Source: PLoS One. 2015 Oct 28;10(10):e0141323. doi: 10.1371/journal.pone.0141323 (PMC4624937; doi:10.1371/journal.pone.0141323)

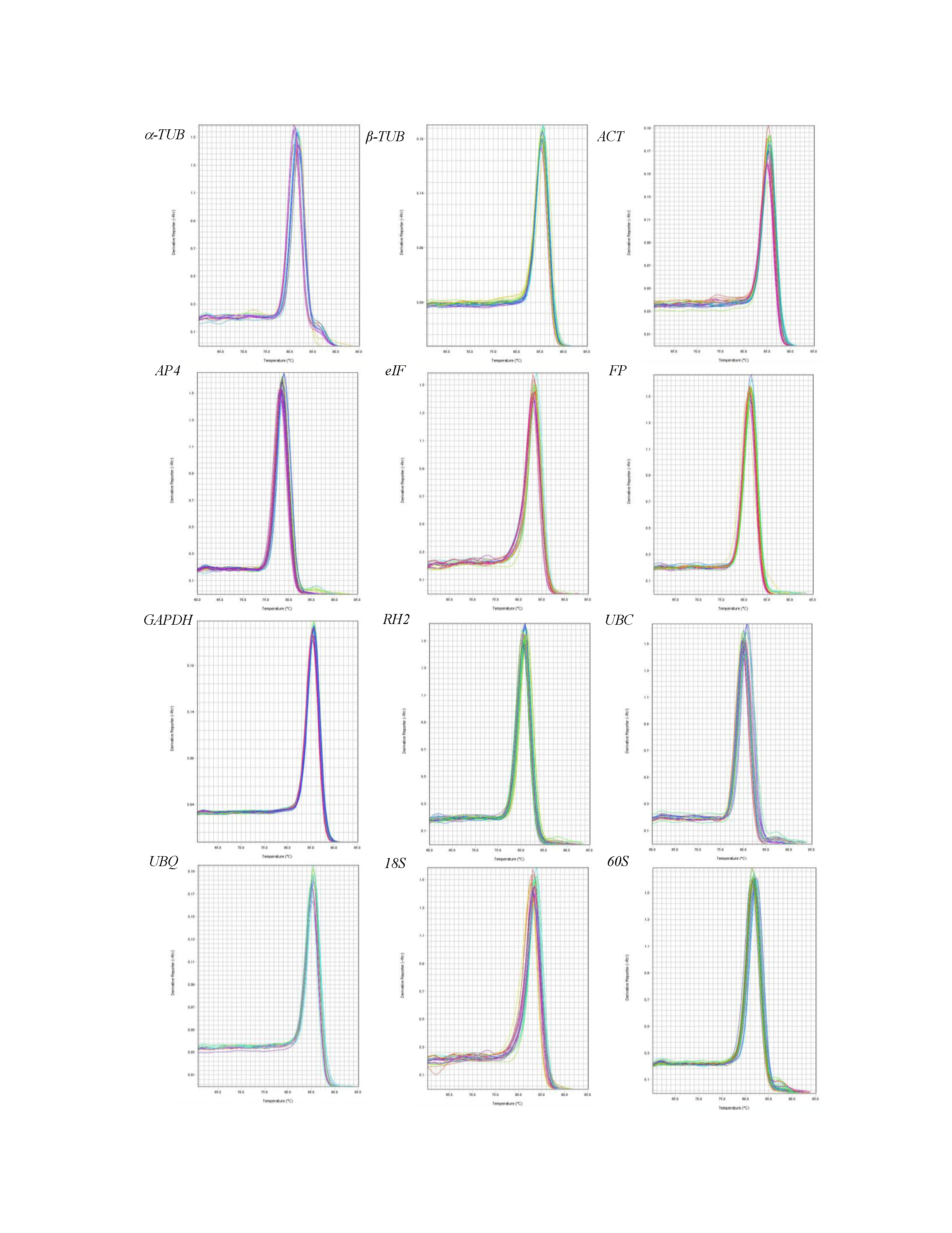

Supplement: S1 Fig — Dissociation curves of the 12 candidate reference genes after the qRT-PCR reactions, all showing a single peak. (TIF) [file pone.0141323.s001.tif]
