# Supplementary material for: Validation of Reference Genes for Accurate Normalization of Gene Expression in Lilium davidii var. unicolor for Real Time Quantitative PCR
Source: PLoS One. 2015 Oct 28;10(10):e0141323. doi: 10.1371/journal.pone.0141323 (PMC4624937; doi:10.1371/journal.pone.0141323)
Supplement: S1 File — (DOC) [file pone.0141323.s002.doc]

1*.α-TUB*: TGGCTTCACAGTCTATCCCTCACCTCAGGTCTCCACATCTGTTGTGGAGCCTTACAACAGTGTGCTCTCCACCCATTCTCTTCTGGAGCACACTGATGTTGCTGTTCTCCTTGACAATGAAGCTATTTACGACATTTGCAGGCGCTCCCTTGATATTGAGCGCCCCACCTACACCAACCTAAACCGTCTGGTTTCACAGGTGATTTCGTCTCTGACGGCATCCCTGAGGTTTGATGGTGCCCTGAATGTTGATGTCACAGAGTTCCAGACCAATCTTGTCCC

2. *β-TUB* :

CTATGACATCTGTTTCCGCACTCTCAAGCTTGCCACCCCTACCTTTGGCGACCTTAATCATCTCATCTCCGCCACCATGAGTGGAGTGACATGCTGCCTTCGCTTCCCTGGTCAGCTCAACTCCGATCTCCGGAAACTAGCTGTTAACCTCATCCCGTTCCCCCGCCTCCACTTCTTCATGGTAGGATTTGCTCCCTTAACCTCGAGAGGCTCCCAACAGTATCGCT

3. *ACT:*

ATCTATGAGGGTTATGCTCTTCCGCATGCCATTCTGAGGCTTGACCTCGCTGGCAGGGATCTGACTGACTCCCTCATGAAAATCCTGACTGAGAGGGGCTACATGTTCACAACTACAGCTGAACGGGAAATTGTCCGTGACATCAAGGAGAAGCTTGCATATGTGGCTCTAGACTATGAGCAGGAGCTAGAAACAGCAAAGAGCAGCTCATCAGTTGAGAAGAATTACGAGCTGCCTGATG

4. *AP4:*

GATGGGGCTTCTTTATACGGTCAGGCAACAGGTTATGGTAGCTCTGCTGGAGGTGTTGTCATACTTGATGATTGTAATTTCCATGAATCTGTACATCTGGACAGTTTTGATGCAGATAGAACTTTGACACTGGTACCTCCAGAGGGAGAGTTTGCTGTAATGA

5. *eIF:*

GATGGGGCTTCTTTATACGGTCAGGCAACAGGTTATGGTAGCTCTGCTGGAGGTGTTGTCATACTTGATGATTGTAATTTCCATGAATCTGTACATCTGGACAGTTTTGATGCAGATAGAACTTTGACACTGGTACCTCCAGAGGGAGAGTTTGCTGTAATGA

6. *FP:*

TCGCCTACATCGCTAACCATGCTGGCTCCCTTCAAACTCTCGAACTACCAAGGTGTGAAATCAGCGATTCGATCGTAGAACAGGTCGCATCAAGACTATCCACTGTCACCTTCTTGGATGTCAGCTACTGTCAAAAGCTCGGTGCCCCTGGTCTTGCGATTATTGGGAA

7. *GAPDH*

GCTGCAAGTTTCAACATTATTCCAAGCAGCACTGGTGCGGCTAAGGCTGTTGGCAAAGTTCTCCCTGCATTGAATGGCAAGCTAACTGGAATGGCTTTCCGTGTTCCCACTGTCGATGTGTCTGTTGTTGATCTCACTGTTAGGCTTGAGAAGGCTGCTACATATGATCAGATCAAGGCTGCTATTAAGGAAGAGTCAGAGGGAAAACTGAAAGGAATTCTTGGTTATACTGATGAGGAT

*8.* *RH2*

CCGAGACCAGTTCGTTCATTTCAAGAAGCTAACTTCCCAGATTACTGTCTCCAGGTGATAGCACAAAGTGGTTTTGTTGAACCAACATCCATCCAATCCCAAGGGTGGCCCATGGCCTTGAAGGGTAGAGATCTAATTGGTATTGCAGAGACTGGTTCCGGGAAGACACTGGCATATCTATTGCCAGGTTTGGTCCATGTTAATGCACAACCTCGCTTAGCCTATGGGGATGGTCCTATTGT

*9.* *UBQ*

TATGGTGGATTATCGGTTTCTACTGGGTATCTGCTGGAGGCCAAGCTTTGACTCATGATGCGCCCCAACTTTACTGGCTTTGTATAGTATTTCTGGCATTTGATGTGTTCTTTGTGGTTTTTTGTGTTGCTTTGGCTTGCGTTATTGGCATTGCTGTTTGCTGCTGCCTACCTTGCATTATTGCAATTTTATATGCTGTGGCAGATCAGCAGGAAGGAGCTTCCGATGAAGACATCCGCCAGCTTCCAAAGTACAAATTTAGAAGAATATGCGATACTGAAAAAGTCTGTGGT

*10.* *UBC*

GAGTGGAGCGTGACCATAATCGGCCCTCCGGATACTCTCTATGATGGGGGCTATTTCAATGCTATCATGAGCTTTCCACCGAATTATCCGAACAGTCCTCCGACTGTTAGGTTTAAGTCTGAGATGTGGCATCCGAATGTTTATCCGGATGGGCGTGTTTGCATATCAATTCTGCATCCACCAG

*11. 18S*

CGTTTCGGGCACGATTTGCGGGGGACGAACGAAACCCCGGCACGGCCTGTGCCAAGGAACATATGTCAGGACGGACGCTCGTCAATGCCTCAGTGGTGGGGCGACGTTCGCTCTCTATCTATACGACTCTCGGCAACGGATATCTCGGCTCTCGCATCGATGAAGAACGTAGCGAAATGCGAT

*12. 60S*

GCAAAGGCTGTCAAAAATCAGGTAGCGGACAACTACTATAGGCCTGATCTGACCAAGGCTGCCCTTGCAAGGCTGAGTGTTGTTCATCGCAGCCTCAAGGTCTCCAAGTCTGGTCCCAAGAAGAAGAACAGGCAGGGCTATTAGTTTGTGGGTTAT
